# Clinical evaluation of usefulness and effectiveness of sitting type continuous passive motion machines in patients with total knee arthroplasty: a study protocol for a single-blinded randomized controlled trial

**DOI:** 10.1186/s12891-022-05507-2

**Published:** 2022-06-10

**Authors:** Byung Chan Lee, Chang Won Moon, Woo Sung Choi, Young Mo Kim, Yong Bum Joo, Da Gyo Lee, Sook Joung Lee, Eun Seok Choi, Jong Hun Ji, Dong Whan Suh, Kang Hee Cho

**Affiliations:** 1 Department of Rehabilitation Medicine, Chungnam National University College of Medicine, 266 Munhwa-ro, Jung-gu, Daejeon, 35015 Korea; 2 Department of Biomedical Institute, Chungnam National University, Daejeon, Korea; 3 Department of Orthopedic Surgery, Chungnam National University College of Medicine, Daejeon, Korea; 4 Catholic University of Korea Industry Academic Cooperation Foundation. The Catholic University of Korea, Seoul, Korea; 5 Department of Rehabilitation Medicine, Daejeon St. Mary’s Hospital, College of Medicine, The Catholic University of Korea, Seoul, Korea; 6 Department of Orthopedic Surgery, Daejeon St. Mary’s Hospital, College of Medicine, The Catholic University of Korea, Seoul, Korea

**Keywords:** Knee Osteoarthritis, Total knee arthroplasty, Continuous passive movement, Range of motion, Post-operative period, Study protocol

## Abstract

**Background:**

Total knee arthroplasty (TKA) is an important management strategy for patients with knee osteoarthritis (OA) refractory to conservative management. Postoperative range of motion (ROM) exercise is important to recover patients’ activities of daily living. Continuous passive motion (CPM) is a machine that provides passive ROM exercises of the knee joint in a pre-defined arc of motion. The short- and long-term effects of CPM exercise are controversial. We hypothesized that the inconsistent results of the CPM exercise are due to poor fitting of CPM machines and measurement errors. This study aims to present a protocol for investigating a new type of CPM machine that could be applied in a sitting position in comparison with the conventional type of CPM machine for patients with unilateral TKAs.

**Methods:**

This study presents the protocol of a prospective, multicenter, single-blinded, three-armed randomized controlled trial (RCT). One hundred and twenty-six patients receiving unilateral TKAs will be recruited at the physical medicine and rehabilitation clinics of two urban tertiary medical hospitals. The patients were randomly divided into three groups with a 1:1:1 allocation. The intervention group will receive two weeks of post-operative rehabilitation using a new type of CPM machine. The control group will receive 2 weeks of post-operative rehabilitation using conventional CPM machines. The third group will receive post-operative rehabilitation with both types of CPM machines. The primary outcome will be the change in the passive ROM of the affected knee joint from baseline to 2 weeks after baseline assessment. The secondary outcomes will be pain and functional measurements, and will include patient-reported outcomes and performance tests surveyed at multiple time points up to 3 months after TKA.

**Discussion:**

This is the first RCT to investigate the effect of a new type of CPM machine. The results of this RCT will determine whether the position of the patients during CPM exercise is important in post-operative rehabilitation protocols after TKAs and will provide evidence for the development of proper rehabilitation guidelines after TKAs.

**Trial registration:**

Clinical Research Information Service of Republic of Korea, KCT0005520, Registered on 21 October 2020, https://cris.nih.go.kr/cris/search/detailSearch.do/21750

**Supplementary Information:**

The online version contains supplementary material available at 10.1186/s12891-022-05507-2.

## Background

Knee osteoarthritis (OA) is a joint disease characterized by the failed repair of joint damage caused by external stress [1]. Symptoms and signs of knee OA include pain, stiffness, and reduced range of motion (ROM), resulting in discomfort and reduced physical activity in older adults.

Several guidelines suggest non-pharmacological and pharmacological treatments and emphasize the importance of maintaining physical fitness and avoiding sedentary lifestyles for patients with OA [2–4]. In cases of refractory knee OA, surgical treatment can be a useful clinical management tool. Among surgical procedures, total knee arthroplasty (TKA) is one of the most successful and has resulted in a substantial increase in quality-of-life gain in patients with refractory arthritis [5].

Continuous passive motion (CPM) machines provide passive ROM exercises of the knee joint in a pre-defined arc of motion. CPM can be used in the early rehabilitation of patients after TKAs and has been suggested to have advantages in regaining ROM of the knee joint in several reports in the 1980s [6, 7]. Recent systematic reviews show controversy in routine use of CPM and its advantages. A Cochrane review on CPM after TKAs was published in 2014. In this review, CPM increased active knee flexion ROM by only 2° compared to the control groups and did not have clinically important short-term effects on pain. CPM reduced the risk of manipulation and adverse events, although the quality of evidence was very low [8]. Yang et al. reported a systematic review on use of CPM after TKAs in 2021, which demonstrated a slight difference in active knee extension at 1 week (mean difference = 3.00°, *P* = 0.019), passive knee extension at 1 week (mean difference = 3.00°, *P* = 0.031), and at 3 months (mean difference = 3.00° *P* = 0.019) compared with the control groups [9].

Many researchers and clinicians do not believe that these slight statistical differences in ROM lead to meaningful clinical outcomes. Moreover, the long-term efficacy of CPM after TKAs has been questioned by many researchers. Some studies showing the superiority of CPM over physical therapy alone applied the regimens with a larger arc of motion [10, 11]. Bible et al. found discrepancies between the pre-defined arc of motion in CPM and the actual arc of knee motion [12]; this can be a potential reason for the controversy in the results of studies using a CPM machine. Therefore, proper fitting and setting of the true knee motion manipulated by CPM can be key factors in post-operative rehabilitation using CPM machines. Moreover, Bible et al. discovered that increasing the angle of inclination of the bed from 30° to 60° enhances the accuracy of the CPM protocol [12].

**Fig. 1:**
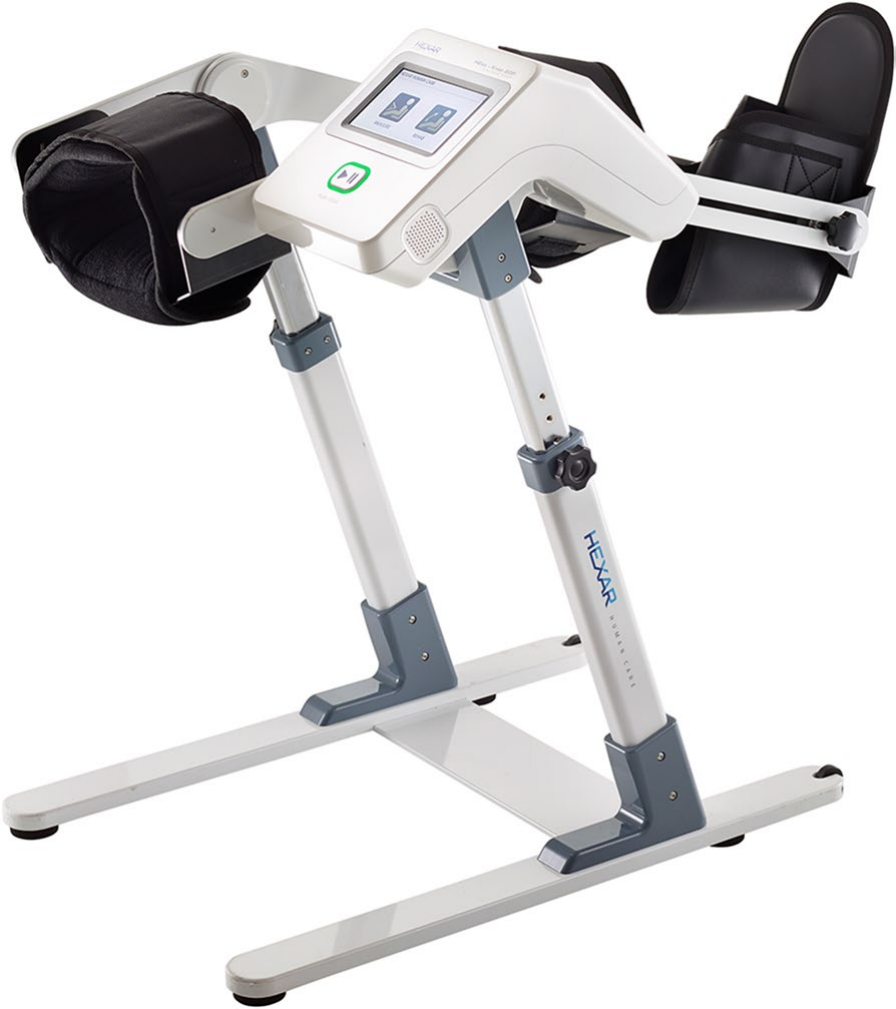
A new type of CPM machine (Resilion K20P®, HEXAR humancare, Korea) will be used in this randomized trial

Therefore, changing the inclination of the bed could be a useful modification of the CPM protocols to enhance effective postoperative rehabilitation. Conventional models of CPM machines are applied in the supine position, and additional bed modifications are required to change the bed tilt angle. However, a CPM machine (Resilion K20P, HEXAR Humancare, Korea, Fig. 1) that can be used in a sitting position has been developed and is currently being applied to patients [13]. However, to the best of our knowledge, there are no reports comparing the effects of CPM between lying and sitting positions in patients receiving TKRs.

We hypothesized that the efficacy of CPM machines in patients receiving TKRs differed according to the position of the patients and that CPM machines applied in the sitting position of patients would be more effective than conventional methods of CPM treatment. Therefore, the purpose of this study was to compare the effect of conventional CPM and CPM treatments applied in the sitting position in patients receiving TKAs and to establish a more suitable rehabilitation strategy using a CPM machine.

## Methods/design

### Overview of research design

**Fig. 2:**
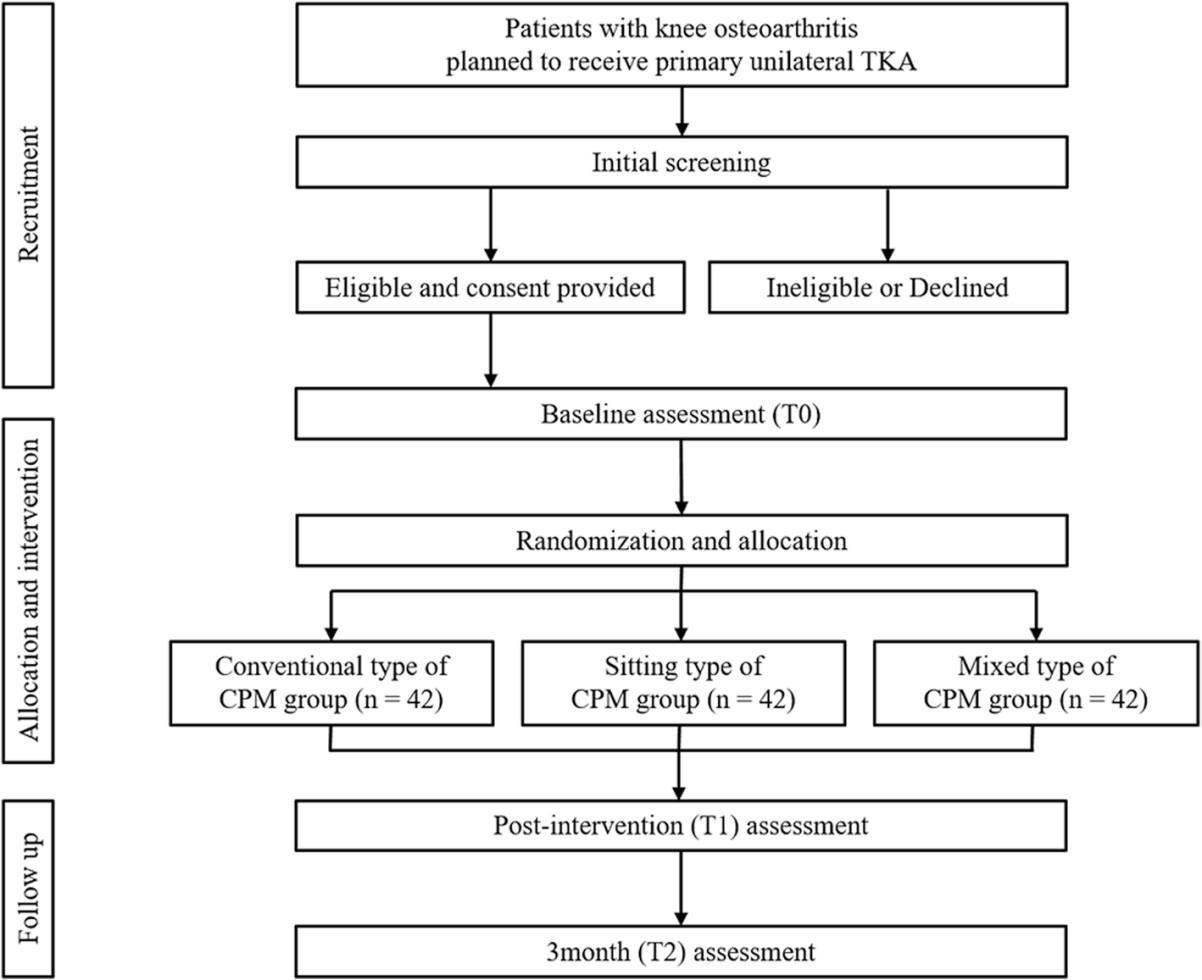
The trial flow diagram

This is a randomized, controlled, three-arm, assessor-blinded trial comparing two types of CPM in acute rehabilitation after unilateral TKA in patients with degenerative knee OA in two tertiary urban medical hospitals. We followed the SPIRIT statement [14] to report the protocol and the SPIRIT Checklist are attached as Additional file 1. Fig. 2 provides an overview of the study’s conduct, review, description, and interpretation.

Written informed consent will be obtained from all the patients. The ethical validity of the trial was assessed and approved by the Institutional Review Boards of Chungnam National University Hospital (CNUH-2020-07-028) and the Catholic University of Korea, Daejeon St. Mary Hospital (DC20DNDI0089). This clinical trial was registered with the Clinical Research Information Service (KCT0005520).

### Participants

#### Eligibility criteria

Eligible participants will be patients scheduled to undergo primary unilateral TKA owing to degenerative changes. Other inclusion criteria will be as follows: 1) age 19 years or older and 2) agreement to participate in this randomized trial.

#### Exclusion criteria

The exclusion criteria will be as follows: 1) age under 60 years old or over 90 years old; 2) cause of arthritis other than degenerative changes (infection, trauma, and inflammatory joint disease); 3) history of previous hip, knee, or ankle orthopedic surgery; 4) history of previous lumbar surgery within 3 months of the study; 5) serious nervous system, cardiovascular diseases, or osteoporosis; 6) coagulation disorders; 7) patients with obesity whose body mass index is more than 35 kg/m^2^, 8) inability to understand the study protocols due to cognitive deficits or language problems; and 9) refusal to participate.

#### Exit criteria

The exit criteria will be as follows: 1) participant request, 2) severe intra- or postoperative complications (i.e., wound complication, fracture, pulmonary embolism, etc.), and 3) intra-treatment side effects.

#### Randomization, allocation, and blinding

Orthopedic surgeons perform an initial screening of all patients who visit the department of orthopedic surgery due to degenerative knee OA. If the patients fulfill the eligibility criteria, they are referred to the principal study coordinator. The principal study coordinator performs the final eligibility assessment and asks for informed consent to enroll in this study. After their consent is obtained, participants are randomly assigned to each group in a 1:1:1 ratio to the conventional type, mixed type, and sitting type CPM groups. Block randomization will be used on groups of 12 to balance relocation into the intervention groups. After the initial allocation, the mixed CPM groups will be re-allocated into two groups. Mixed groups will receive combined interventions with two types of CPM machines and consist of two subgroups. One of the subgroups will receive intervention using the conventional type of CPM, followed by the sitting type of CPM, and the other subgroup will recieve intervention in the opposite order. This trial will be blinded to the assessor; after randomization, the allocation of participants will be confidentially managed by the principal study coordinator. All assessments will be conducted by a single trained physiotherapist in each hospital who is experienced in post-operative rehabilitation treatment and will be blinded to group allocation. Due to the nature of the study, participants will not be blinded and will be instructed not to communicate with any other researcher or physiotherapists about their interventions to minimize potential bias. If participants would like to discuss their rehabilitation program during the intervention, they will be able to do so with the unlabeled study coordinator.

#### Intervention protocol

**Fig. 3:**
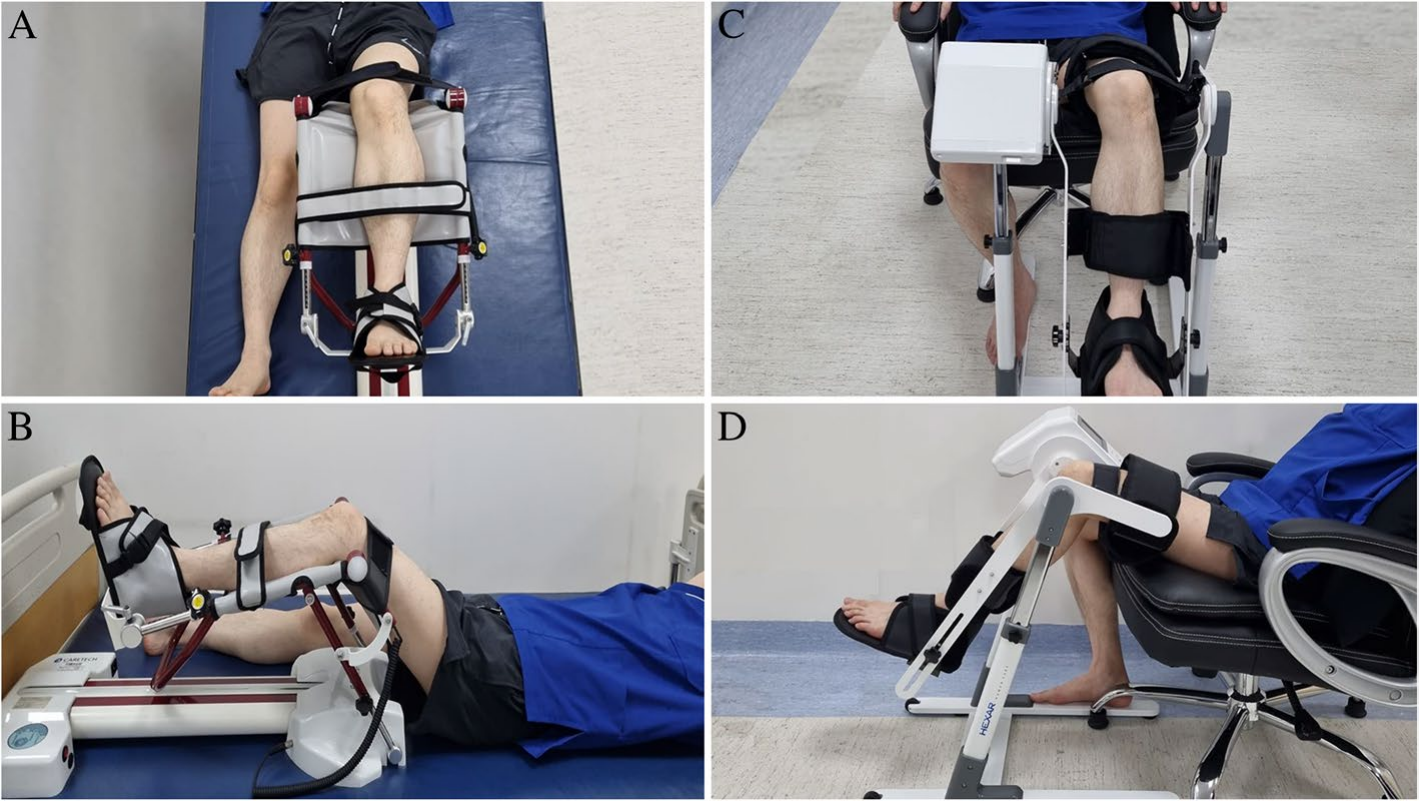
CPM machine set up. A conventional type of CPM machine (RCF1121®, CARETECH, Korea) (A-B), New type of CPM machine (Resilion K20P®, HEXAR humancare, Korea) (C-D) Each CPM machine will be applied to the participants according to the manufacturer's instruction

All participants will undergo primary unilateral TKA at the Department of Orthopedic Surgery. The day before the surgery, all participants will be admitted to the ward. On the day of admission, basal demographics and outcomes will be collected by the assessor. All participants will be instructed on the interventions and post-operative exercise programs. The participants will start the intervention program on postoperative day 3, the day after the removal of the surgical site drainage system. Two CPM systems (sitting type and conventional type) will be applied to the allocated group of participants (Fig. 3). The pre-defined arc of the angles in the CPM machines will be determined according to the ROM of the knee joint measured before each intervention. The assessor will measure the maximal passive ROM of the knee joint by using a goniometer. The ROM measurements will be taken in all three positions: supine, sitting, and prone. The setting arc of the angles of the CPM machines will be determined by adding 10° to the highest measured values in the ROM in the three positions. On the first day, interventions will be applied twice daily for 30 min. From the second day to the end of the intervention, CPM machines will be applied twice a day for 1 h in each session, for a total of 2 h per day. All participants were mobilized as tolerated using a walker or crutches. Conventional CPM will be performed using the RCF1121® (CARETECH, Gumi-si, Gyeongsangbuk-do, Korea). The CPM machines used in the sitting position will be Resilion K20P® (HEXAR Humancare, Ansan-si, Gyeonggi-do, Korea). Each group of participants will receive 10 sessions of intervention using the allocated CPM machines. The group of participants planned using both types of CPM machines will consist of two sub-groups, one of which will receive intervention for 5 consecutive days using the conventional type of CPM, followed by 5 days of intervention using the sitting type of CPM, and the other sub-group will receive intervention in the opposite order.

#### Outcome measurement and participants’ timeline

**Fig. 4:**
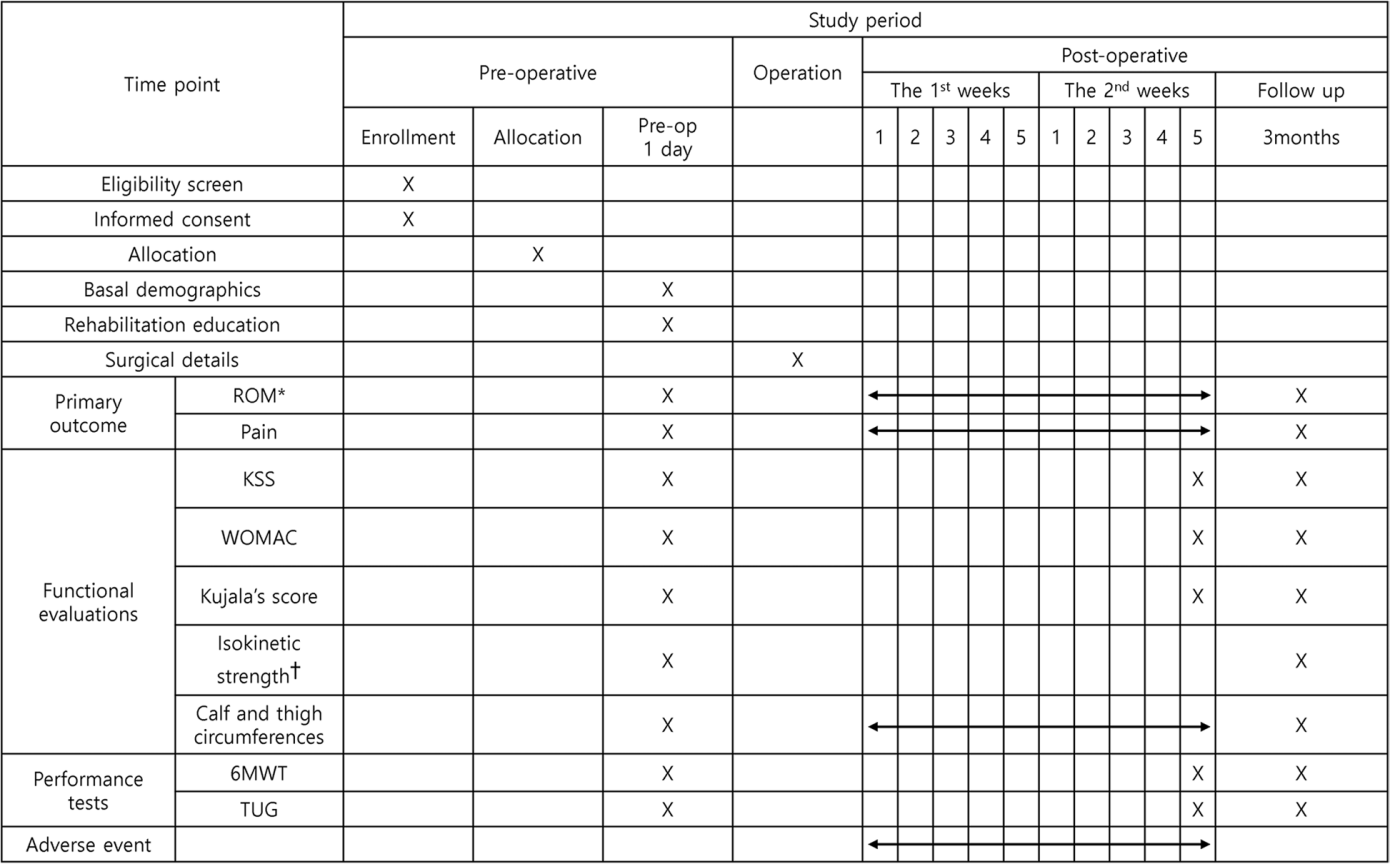
The patients' schedule of trial enrollment, interventions, and assessment. ROM, Range of motion; KSS, Knee society score; WOMAC, Western Ontario and McMaster Universities Osteoarthritis Index; 6MWT, 6-minute walking test; TUG, Timed up and go test. ^*^Both active and passive ROMs of knee flexion and extension will be measured. ^†^Isokinetic strength of knee flexion and extension measured at 60°/sec angular velocity using an isokinetic dynamometer (Systemic 4 Pro, Biodex Medical System, USA). ^‡^ Mixed-type CPM group only

The primary outcomes are ROM of the knee joint at the end of the intervention and 3 months after TKA. The secondary outcomes are pain, and functional measurements included patient-reported outcomes and performance tests conducted at multiple time points up to 3 months after TKA. During the trials, the satisfaction questionnaire (additional file) will be administered only to participants of the mixed-type CPM group. Participants will be screened for adverse events from the time of informed consent to 3-months postoperatively. The study assessment schedule is illustrated in Fig. 4. Demographic details, including sex, age, height, weight, affected side, and medical history, will be surveyed preoperatively by the research delegate.

### Primary outcome measurement

#### Range of motion in knee joint

A blinded physiotherapist will measure active and passive ROM in the affected knee joint using a digital inclinometer (Dualer IQ electronic dual inclinometer system; JTECH Medical, UT, USA). Passive ROM will be measured with the patients in the supine, sitting, and prone positions. When evaluating the passive ROM, the assessor will mobilize the knee joint to the end of the range of applied force of 2 kgf using a handheld dynamometer (MicroFET2, Hoggan Health Industries, UT, USA). Active and passive ROM will be evaluated at three time points: the day before the surgery, after the intervention, and 3-months postoperatively.

### Secondary outcome measurement

#### Pain

A numeric rating scale will be used to evaluate the pain of the participants during and at the end of the intervention, and 3-month follow-up. The numeric rating scale is an 11-point parameter for describing the severity of reported pain, ranging from 0 (no pain) to 10 (intractable pain) [15]. Participants will be asked about their average perceived pain intensity in the past day.

### Patients reported outcomes

Patient-reported outcomes will be measured using the Knee Society Score (KSS), Western Ontario and McMaster Universities Osteoarthritis Index (WOMAC), and Kujala Patellofemoral score [16–18].

The KSS is an objective scoring system to assess patient reporting outcomes in osteoarthritis, especially after TKA. The KSS is easy to obtain and concise. The Korean version of the KSS was validated in 2017 by Kim et al. [16], and participants will be assessed using this version.

The WOMAC score was developed by Bellamy et al. [19] and is widely applied to evaluate the status of lower-limb osteoarthritis. The WOMAC questionnaire consists of 24 questions in three domains. Pain (five items), stiffness (two items), and function (17 items) are evaluated on a total scale of 0–100; higher scores indicate greater disability. The Korean version of WOMAC was validated in 2001, and patients will be evaluated using this version [17].

The Kujala patellofemoral score will also be calculated. The Kujala patellofemoral score consists of 13-items with categories related to various levels of functional knee problems. All responses are summed to provide an overall index ranging from 0 to 100 points, with 100 points representing no disability [18].

Satisfaction questionnaires (Additional file 2) will be administered to participants in the mixed-type CPM group at the end of the intervention to compare conventional and new types of CPM machines.

### Swelling

Lower limb swelling after TKAs is a normal response to surgical trauma [20] and may impair postoperative mobilization and the effects of postoperative rehabilitation. Swelling after TKAs was significantly more pronounced in the operated limb and swelling above the knee was more severe than that below the knee [20]. Two randomized trials reported the effect of CPM treatment on the statistically significant reduction in knee swelling after TKAs [21, 22]. We will measure the extent of swelling of the limb through circumference taken 10 cm superior to the upper patellar pole and 10 cm inferior to the lower patellar pole during and at the end of the intervention and 3-month follow-up.

### Gait ability

The Timed Up and Go test (TUG) and 6-minute walking test will be conducted to assess the functional gait ability of the participants. During the TUG test, the patient will be asked to get up from the chair, walk 3 m, return, and sit again. Assessment will be timed during the test. This test has long been used to measure the walking ability of the elderly and is known to be reliable and correlates with the Berg balance test and walking speed [23]. The 6-minute walk test is widely used to assess physical exercise capacity in frailty or sarcopenia in the elderly [24]. The patient will be asked to walk as far as possible along the 30 m corridor for 6 min, and the distance traveled will be measured in meters.

### Isokinetic strength measurement

Knee osteoarthritis can cause weakness of the periarticular skeletal muscles, mainly in the knee extension group. This weakness of knee extension slows the gait speed of the patients, and therefore, is an important indicator of gait [25]. After TKA, isokinetic measurements using an isokinetic dynamometer are known to be reliable, easy to perform, accurate, and safe [26]. Peak torques of the knee flexors and extensors of both sides will be measured during concentric contraction at 60°/sec angular velocity using an isokinetic dynamometer (Systemic 4 Pro; Biodex Medical System, New York, NY, USA).

### Sample size calculation

The sample size was calculated based on the data of a preliminary pilot study conducted by our group. The number of participants in the pilot study was 16 (conventional CPM group: 6, sitting-type CPM group: 6, mixed-type CPM group: 4). The primary hypothesis was that there would be a significant difference in the passive ROM before and after treatment between the groups. The means ± standard deviations of passive ROM of the knee joint after treatment were 105.2±14.7, 100.0±26.5, 92.5±8.9 in conventional CPM group, sitting type CPM group, and mixed-type CPM groups respectively, in the preliminary pilot study.

Thirty-five people per group were required for a significance level of 5% (a significance level of 0.05/4 × 100% of each hypothesis) and 80% statistical power. Assuming an expected dropout rate of 20%, at least 42 participants per group will be required.

### Data management

All participant data will be collected and processed by members of the research team and stored securely on a platform only accessible to the research team. The backup database will be regularly updated. The anonymized dataset will be made available upon request from the corresponding authors. No intermediate analysis will be carried out before the study's conclusion.

### Statistical analysis

Descriptive statistics will be used to calculate subject demographics. The Kolmogorov–Smirnov test will be used to evaluate the data distribution. We will also conduct an intention-to-treat analysis. One-way analysis of variance or Kruskal–Wallis test will be used to compare baseline values between groups. After the study was completed, the method for dealing with missing data will be determined based on the distribution of the data.

A mixed-effects model or generalized estimating equations will be used to investigate the effects of the interventions and differences between groups for secondary outcomes, with one between-subject factor (group: intervention and control) and one within-subject factor (group: intervention and control) (evaluation time: baseline, end-of-intervention, and 3-month follow-ups). All data will be analyzed using IBM SPSS Statistics (version 26.0; Armonk, New York, USA), and a significance level of 5% will be set with a 95% confidence interval.

### Predicted adverse events and monitoring

The potential risk level, as reviewed by the institutional review board and the principal investigator, is minimal. To prevent and manage adverse events, participants can call researchers at any time if they have any questions or problems. The treating physiotherapist will report each adverse event to the primary study coordinator. In addition, if the patient's pain continues to worsen during exercise or if these symptoms do not resolve before the next exercise session, the physiotherapists will report to the primary study coordinator, and the primary investigator can refer to the orthopedic specialist. The number and seriousness of the adverse events will be reported.

### Ethics and dissemination

The institutional review boards of the two hospitals approved all study procedures (approval numbers: CNUH-2020-07-028 and DC20DNDI0089). This clinical trial was registered with the Clinical Research Information Service (KCT0005520). If significant protocol changes are made, the primary investigator will inform the coordinating investigators and trial participants, as well as the institutional review board. During the study, personal information about the recruited participants will be gathered and shared with the clinic (Daejeon St. Mary Hospital), and only the research team will have access to it. After the study is completed, all personal information will be kept for three years before being destroyed. The trial results will be published in the journal and the report of results will be posted on the funding institute’s site for the public, participants, and healthcare professionals.

## Discussion

We present a protocol for a multicenter, randomized clinical trial comparing the conventional and sitting types of CPM in patients with knee osteoarthritis receiving unilateral TKA. To our knowledge, this randomized trial is the first to investigate the effect of CPM training in the sitting position in patients receiving unilateral TKAs. In addition, we will apply objective methods of measuring the ROMs by applying uniform forces (2 kgf) using a hand-held dynamometer.

In a previous systematic review of TKA, significant improvements in WOMAC and KSS from the baseline were observed in pain, function, and total score [27]. In the United States, more than 700,000 TKAs are performed annually, and Kurtz et al. projected that this number will reach 3.5 million by 2030 [28]. In Korea, TKAs increased by approximately 68% between 2010 and 2018, and the number of TKAs is expected to increase by approximately 53% by 2030 in a projection model [29].

However, 15–20% of patients suffer from post-operative long-term discomfort after TKAs [30, 31]. One of the reasons for remnant discomfort after TKAs is the limited postoperative ROM of the knee joint. The achievement of at least 110° of knee flexion has been known as the optimal treatment goal and is sufficient to provide a satisfactory function for most patients for most activities of daily living [32, 33]. Therefore, to regain the satisfactory outcomes after TKAs, advised and active mobilization of the knee joint should be incorporated into the postoperative rehabilitation protocols.

Therefore, the importance of postoperative rehabilitation has been emphasized. However, despite the abundance of post-operative rehabilitation modalities and strategies, there are no consistent or widely implemented guidelines for rehabilitation following TKR. CPM is not routinely recommended because of inconsistent results in previous studies [8, 9].

The authors hypothesized that these inconsistent results were due to the improper fitting of CPM machines with patients and measurement error of ROM between studies. Good to excellent inter-tester reliability and intra-tester reliability have been reported for measuring the ROM in the native knee using a simple goniometer [34, 35]. However, measuring the knee joint ROM in patients after TKA shows that 22% of the goniometer measurements had more than 5° differences from the true degree of knee flexion [36]. Therefore, we decided to apply more objective tools to measure the ROMs using a digital inclinometer and a hand-held dynamometer. Therefore, this randomized trial will be conducted using more objective measurement tools and exact prescribed pre-defined arc ranges than previous trials using CPM machines.

In conclusion, the randomized trial will provide evidence of using a new type of CPM machine applied in the sitting position to incorporate the use of CPM into post-operative rehabilitation guidelines in patients receiving TKAs.

## Trial status

Protocol version number V1.1, 2 April 2021.

Patient recruitment began in 3 April 2021; a total of 68 patients have been recruited across two institutions, and the approximate date when recruitment will be complete is 31 November 2022. The trial is currently in follow-up until March 2023.

## Supplementary Information

**Additional file 1.**

**Additional file 2.**

## Data Availability

Not applicable.

## Abbreviations

OA: Osteoarthritis
TKA: Total knee arthroplasty
CPM: Continuous passive motion
ROM: Range of motion
KSS: Knee Society Score
WOMAC: Western Ontario and McMaster Universities Osteoarthritis Index
TUG: Timed up and go test
