# Supplementary material for: Clinical evaluation of usefulness and effectiveness of sitting type continuous passive motion machines in patients with total knee arthroplasty: a study protocol for a single-blinded randomized controlled trial
Source: BMC Musculoskelet Disord. 2022 Jun 10;23:565. doi: 10.1186/s12891-022-05507-2 (PMC9188049; doi:10.1186/s12891-022-05507-2)
Supplement: Supplementary file 2 — Additional file 2. [file 12891_2022_5507_MOESM2_ESM.docx]

**Passive joint exercise equipment satisfaction questionnaire**

This questionnaire is intended to survey your level of satisfaction with passive joint exercise equipment and will be used for future product improvement ideas. If you have any questions regarding the questionnaire, please feel free to contact us.

Thank you for your comments.

| **Response** | | | **Answer** | | | | |
| --- | --- | --- | --- | --- | --- | --- | --- |
| Convenience | 1 | Was it convenient to move from the wheelchair to another place in order to use the equipment? | Very Satisfied | Satisfied | Neutral | Dissatisfied | Very Dissatisfied |
|  | 2 | Was it easy to mount the equipment around the legs? | Very Satisfied | Satisfied | Neutral | Dissatisfied | Very Dissatisfied |
|  | 3 | Was the shape of the equipment suitable to be worn on the legs? | Very Satisfied | Satisfied | Neutral | Dissatisfied | Very Dissatisfied |
|  | 4 | Were there any inconveniences when tying the legs with the strings? | Very Satisfied | Satisfied | Neutral | Dissatisfied | Very Dissatisfied |
|  | 5 | Were there any inconveniences when removing the equipment after treatment? | Very Satisfied | Satisfied | Neutral | Dissatisfied | Very Dissatisfied |
|  | 6 | After use of the equipment, was it convenient to return to the wheelchair? | Very Satisfied | Satisfied | Neutral | Dissatisfied | Very Dissatisfied |
| Effectiveness | 7 | Was the speed of the moving equipment satisfactory during the treatment? | Very Satisfied | Satisfied | Neutral | Dissatisfied | Very Dissatisfied |
|  | 8 | Did the equipment and the legs move well together in accordance without falling apart during treatment? | Very Satisfied | Satisfied | Neutral | Dissatisfied | Very Dissatisfied |
|  | 9 | Were you satisfied with the intensity of the exercise during treatment? | Very Satisfied | Satisfied | Neutral | Dissatisfied | Very Dissatisfied |
|  | 10 | Were there any uncomfortable areas, other than the legs, during treatment? | Very Satisfied | Satisfied | Neutral | Dissatisfied | Very Dissatisfied |
|  | 11 | If there were any inconveniences, which parts were uncomfortable during treatment? | Please write freely (ex. thighs, back etc.) | | | | |
| Satisfaction | 11 | Do you feel a decrease in edema and swelling after using this device? | Very Satisfied | Satisfied | Neutral | Dissatisfied | Very Dissatisfied |
|  | 12 | Do you feel that your joint range of motion has increased after using this equipment? | Very Satisfied | Satisfied | Neutral | Dissatisfied | Very Dissatisfied |
|  | 13 | Are you satisfied with the overall treatment using this equipment? | Very Satisfied | Satisfied | Neutral | Dissatisfied | Very Dissatisfied |

**Passive joint exercise equipment satisfaction questionnaire**

This questionnaire is intended to survey your level of satisfaction with passive joint exercise equipment and will be used for future product improvement ideas. If you have any questions regarding the questionnaire, please feel free to contact us.

Thank you for your comments.

| **Question** | | | **Answer** | |
| --- | --- | --- | --- | --- |
| Convenience | 1 | Which device was more convenient to move from the wheelchair to another place in order to use the equipment? | Sitting device | Device for lying down |
|  | 2 | Which device was easier to mount the equipment around the legs? | Sitting device | Device for lying down |
|  | 3 | Which device had a more suitable shape to be worn on the legs? | Sitting device | Device for lying down |
|  | 4 | Which device was more inconvenient when fixing the legs onto the device (using strings, straps, buckles)? | Sitting device | Device for lying down |
|  | 5 | Which device was more convenient to remove after treatment? | Sitting device | Device for lying down |
|  | 6 | Which device was more convenient to return to the wheelchair after treatment? | Sitting device | Device for lying down |
| Effectiveness | 7 | Which device had a more satisfactory speed during treatment? | Sitting device | Device for lying down |
|  | 8 | During treatment, which device stabilized the legs with the equipment more without falling apart? | Sitting device | Device for lying down |
|  | 9 | Which device had a more satisfactory level of exercise intensity? | Sitting device | Device for lying down |
|  | 10 | Which device had fewer inconveniences and uncomfortable areas during treatment? | Sitting device | Device for lying down |
| Satisfaction | 11 | Which device seemed to reduce swelling and edema more? | Sitting device | Device for lying down |
|  | 12 | Which device seemed to increase your joint's range of motion after treatment? | Sitting device | Device for lying down |
|  | 13 | Overall, which device was more satisfactory? | Sitting device | Device for lying down |
